# Altering sphingolipid composition with aging induces contractile dysfunction of gastric smooth muscle via K_C__a_1.1 upregulation

**DOI:** 10.1111/acel.12388

**Published:** 2015-08-20

**Authors:** Shinkyu Choi, Ji Aee Kim, Tae Hun Kim, Hai‐yan Li, Kyong‐Oh Shin, Yong‐Moon Lee, Seikwan Oh, Yael Pewzner‐Jung, Anthony H. Futerman, Suk Hyo Suh

**Affiliations:** ^1^ Department of PhysiologyMedical SchoolEwha Womans UniversitySeoulKorea; ^2^ Department of Molecular MedicineMedical SchoolEwha Womans UniversitySeoulKorea; ^3^ Department of Internal MedicineMedical SchoolEwha Womans UniversitySeoulKorea; ^4^ College of Pharmacy and MRCChungbuk National UniversityChongjuKorea; ^5^ Department of Biological ChemistryWeizmann Institute of ScienceRehovotIsrael

**Keywords:** aging, Ca^2+^‐activated K^+^ channel, ceramide synthases, contractile dysfunction, smooth muscle, sphingolipids

## Abstract

K_C__a_1.1 regulates smooth muscle contractility by modulating membrane potential, and age‐associated changes in K_C__a_1.1 expression may contribute to the development of motility disorders of the gastrointestinal tract. Sphingolipids (SLs) are important structural components of cellular membranes whose altered composition may affect K_C__a_1.1 expression. Thus, in this study, we examined whether altered SL composition due to aging may affect the contractility of gastric smooth muscle (GSM). We studied changes in ceramide synthases (CerS) and SL levels in the GSM of mice of varying ages and compared them with those in young CerS2‐null mice. The levels of C16‐ and C18‐ceramides, sphinganine, sphingosine, and sphingosine 1‐phosphate were increased, and levels of C22, C24:1 and C24 ceramides were decreased in the GSM of both aged wild‐type and young CerS2‐null mice. The altered SL composition upregulated K_C__a_1.1 and increased K_C__a_1.1 currents, while no change was observed in K_C__a_1.1 channel activity. The upregulation of K_C__a_1.1 impaired intracellular Ca^2+^ mobilization and decreased phosphorylated myosin light chain levels, causing GSM contractile dysfunction. Additionally, phosphoinositide 3‐kinase, protein kinase C_ζ_, c‐Jun N‐terminal kinases, and nuclear factor kappa‐B were found to be involved in K_C__a_1.1 upregulation. Our findings suggest that age‐associated changes in SL composition or CerS2 ablation upregulate K_C__a_1.1 via the phosphoinositide 3‐kinase/protein kinase C_ζ_/c‐Jun N‐terminal kinases/nuclear factor kappa‐B‐mediated pathway and impair Ca^2+^ mobilization, which thereby induces the contractile dysfunction of GSM. CerS2‐null mice exhibited similar effects to aged wild‐type mice; therefore, CerS2‐null mouse models may be utilized for investigating the pathogenesis of aging‐associated motility disorders.

**Abbreviations**
[Ca^2+^]_i_: intracellular Ca^2+^ concentration
CerS: ceramide synthase(s)
IBTx: iberiotoxin
ICC: interstitial cells of the Cajal
K_Ca_1.1: large conductance Ca^2+^‐activated K^+^ channels
MLC: myosin light chain
NF‐κB: nuclear factor kappa‐B
*NPo*: the open probability times the number of channels in the patch
p21^*CIP*^: cyclin‐dependent kinase inhibitor p21
PGF_2α_: prostaglandin F_2α_
p‐JNK: phosphorylated JNK
PKI: PKC_ζ_ pseudosubstrate inhibitor
p‐MLC: phosphorylated MLC
p‐p85: phosphorylated p85
p‐PKC_ζ_: phosphorylated PKC_ζ_
S1P: sphingosine 1‐phosphate
SLs: sphingolipids
SMCs: smooth muscle cells
VOCCs: voltage‐operated Ca^2+^ channels

## Introduction

Aging causes structural changes in the gastrointestinal tract, which cause a significant decline in gastrointestinal function and increase the prevalence of several gastrointestinal motor disorders such as dyspepsia and constipation. Both a decrease in the number and volume of interstitial cells of Cajal (ICC; Gomez‐Pinilla *et al*., 2011) and the degeneration of the enteric nervous system of the gastrointestinal tract, such as the myenteric plexus, occur in an age‐related manner (El‐Salhy *et al*., 1999; Phillips & Powley, 2001). As the ICC and the enteric nervous system play key roles in the control of gastrointestinal motility, the decrease in ICC and the degeneration of the enteric nervous system may contribute to changes in gastrointestinal motility with aging; however, little is known about the effect of aging on smooth muscle.

Gastric smooth muscle (GSM) generates regular contractions, which are triggered by long‐lasting waves of depolarization, or slow waves. The ICC generate slow waves (Hirst & Edwards, 2006) and thereby depolarize nearby smooth muscle cells (SMCs). The depolarization activates voltage‐operated Ca^2+^ channels (VOCCs), and Ca^2+^ entry through VOCCs increases intracellular Ca^2+^ concentration ([Ca^2+^]_i_), which induces smooth muscle contraction. Intracellular Ca^2+^ then activates K_Ca_1.1, which causes the hyperpolarization of SMCs. The hyperpolarization inactivates the VOCCs and thereby induces smooth muscle relaxation by inhibiting Ca^2+^ entry through the VOCCs. Thus, VOCCs and K_Ca_1.1 act as important regulators of gastrointestinal motility by modulating membrane potential and Ca^2+^ influx, and changes in these ion channels greatly affect the contractility of smooth muscle. As the level of K_Ca_1.1 is increased with advancing age (Tricarico *et al*., 1997; Oshiro *et al*., 2005), K_Ca_1.1 upregulation might contribute to the development of age‐associated gastrointestinal motility disorder.

Many different types of ion channels localize to cholesterol and sphingolipid (SL)‐enriched regions of the plasma membrane, known as lipid microdomains or rafts (Dart, 2010). As SLs are important structural components of cellular membranes, they might affect ion channel gating and expression on the cell surface. The short‐acyl chain ceramide analog, C6‐ceramide, has been reported to modulate HERG K^+^ channel gating by causing its translocation into caveolin‐enriched lipid rafts (Ganapathi *et al*., 2010) and to induce the channel downregulation via ubiquitin‐mediated lysosomal degradation (Chapman *et al*., 2005). C6‐Ceramide also inhibited voltage‐gated K^+^ currents and induced vasoconstriction in rat and human pulmonary arteries (Moral‐Sanz *et al*., 2011). Ceramide, the backbone of all complex SLs, is synthesized by the *N*‐acylation of a sphingoid long‐chain base by a family of six ceramide synthases (CerS; Levy & Futerman, 2010), each generating ceramides with a different acyl chain length. C18‐ceramide is generated by CerS1 and CerS4, and C16‐ceramide is generated by CerS5 and CerS6. CerS2 generates very long acyl chain (C22 and C24) ceramides. Ceramides can also be formed by the degradation of complex SLs, such as sphingomyelin or glycosphingolipids. Ceramides can function as a second messenger in a variety of cellular events, including apoptosis and differentiation (Perry & Hannun, 1998; Gulbins, 2003), and the effects of ceramides may vary according to its acyl chain length (Mesicek *et al*., 2010). However, little is known about whether the aging process alters SL composition in cells and thereby affects ion channels to cause gastrointestinal motility disorder.

In this study, we hypothesized that altered SL composition due to aging may contribute to the development of contractile dysfunction of GSM via K_Ca_1.1 upregulation. To test this hypothesis, we measured age‐dependent changes in levels of SLs, K_Ca_1.1 expression, phosphorylated myosin light chain (p‐MLC), and contractility in wild‐type (WT) GSM, and compared these levels with those induced by CerS2 ablation in CerS2‐null mice, which were unable to synthesize very long acyl chain ceramides and contained increased levels of long acyl chain (C16 and C18) ceramides. Importantly, we found that levels of C16‐ and C18‐ceramides, sphinganine, sphingosine (SP), and sphingosine 1‐phosphate (S1P) were increased and levels of C22, C24:1, and C24 ceramides were decreased in the GSM of aged WT and young CerS2‐null mice. It was found that altered SL composition upregulated K_Ca_1.1 via the phosphoinositide 3‐kinase (PI3K)/protein kinase C_ζ_ (PKC_ζ_)/c‐Jun N‐terminal kinases (JNK)/nuclear factor kappa‐B (NF‐κB) pathway in GSM. K_Ca_1.1 upregulation impaired Ca^2+^ mobilization and thereby inhibited myosin light chain (MLC) phosphorylation, which caused contractile dysfunction in GSM. We suggest that altering the SL composition induces contractile dysfunction via K_Ca_1.1 upregulation in aged GSM, and CerS2‐null mice allow us to investigate age‐associated contractile dysfunction in GSM.

## Results

### SL composition is altered in an age‐dependent manner

**Figure 1:**
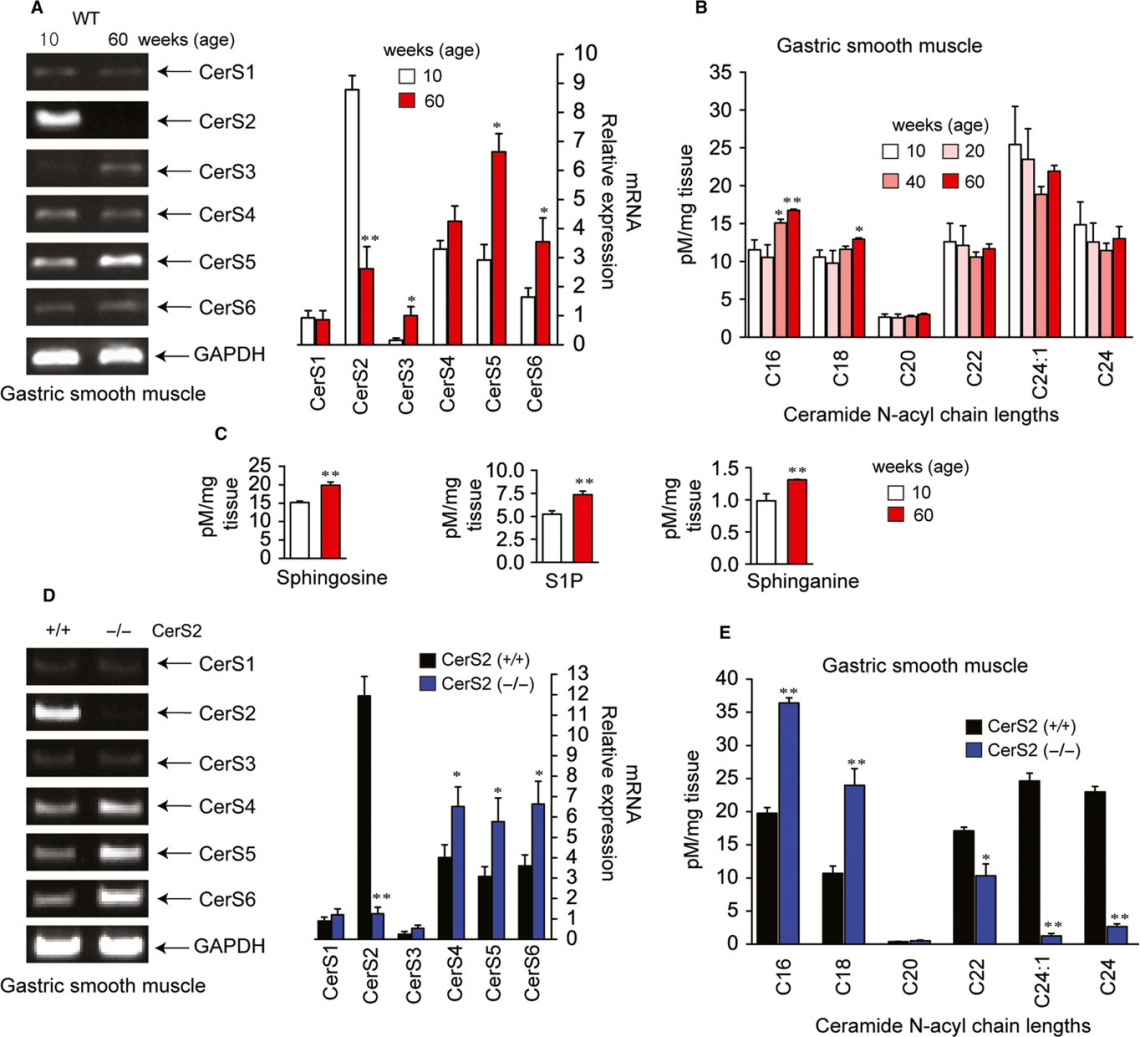
Changes in levels of ceramide synthase(s) (CerS) and sphingolipids (SLs) in gastric smooth muscle (GSM) due to aging or CerS2 ablation. Levels of CerS1–CerS6 mRNA (A,D) and levels of SLs (B,C,E) were measured in GSM of WT mice at different ages (A–C), or of 25‐week‐old CerS2‐null and WT mice (D,E). Blots are representative of three experiments, and the results have been normalized to GAPDH levels. The data are quantified from a set of three experiments. *P < 0.05, **P < 0.01 vs. 10‐week‐old (A–C) or 25‐week‐old (D,E) WT.

We first determined the levels of CerS mRNA in GSM from mice of different ages, from 10 to 60 weeks old. Compared to the GSM from young (10 weeks old) WT mice, the levels of CerS4‐CerS6 mRNA were significantly greater, and CerS2 levels were significantly smaller, in GSM from aged (60 weeks old) WT mice (Fig. 1A). LC‐MS/MS analysis of SL levels exhibited that the levels of C16‐ and C18‐ceramides, SP, S1P, and sphinganine were significantly greater in the GSM from aged mice (Fig. 1B,C), demonstrating that SL composition varies depending on age. As CerS2 levels were significantly smaller in aged mice, we examined whether similar alterations in CerS and SL composition occur in CerS2‐null mice. In the GSM of young (25 weeks old) CerS2‐null mice, levels of CerS4‐CerS6 mRNA were significantly higher compared with those in age‐matched WT mice, whereas the levels of CerS1 and CerS3 were not affected (Fig. 1D). The levels of C16‐ and C18‐ceramides were significantly higher, and the levels of C22, C24:1, and C24 ceramides were markedly smaller in the GSM of CerS2‐null mice (Fig. 1E). The levels of C20‐ceramide were very low in the GSM of both WT and CerS2‐null mice. Similar changes in CerS and ceramide composition were found in primary cultured gastric SMCs (Fig. S1, Supporting information). A previous study reported that levels of SP and sphinganine are increased in CerS2‐null mice (Pewzner‐Jung *et al*., 2010). These results suggest that aging and CerS2 ablation induce similar change in SL composition.

### K_Ca_1.1 upregulation without affecting single‐channel activity

**Figure 2:**
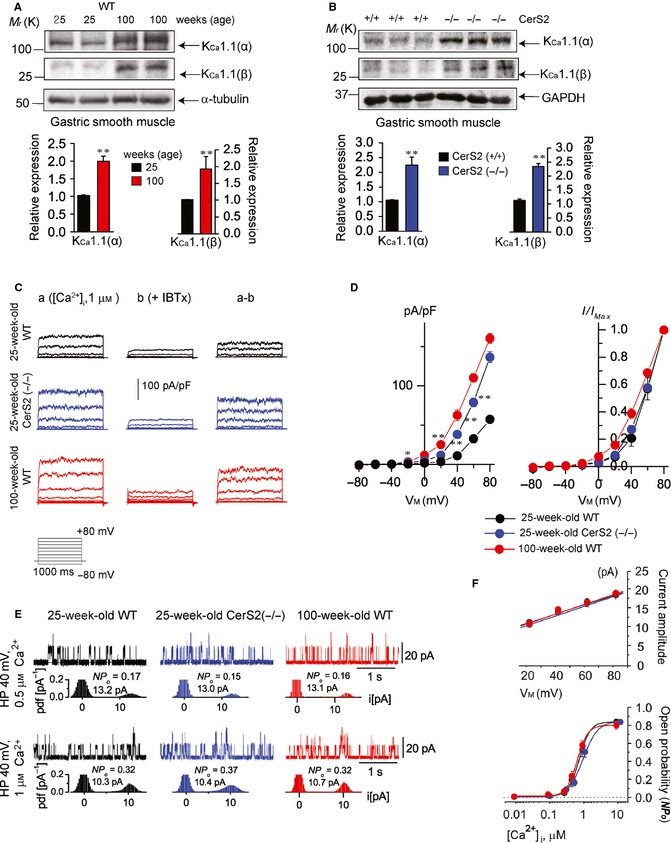
Increased K_C__a_1.1 expression on cell membrane due to aging or CerS2 ablation. (A,B) K_C__a_1.1 protein levels (α‐ and β‐subunits) were examined in gastric smooth muscle of WT mice at different ages (A), and of 25‐week‐old CerS2‐null and WT mice (B). Blots are representative of 4 experiments. Results were normalized to GAPDH or α‐tubulin levels. (C) Representative Ca^2+^‐activated currents in the absence (a) and presence (b) of iberiotoxin (IBTx; 300 nm) in gastric smooth muscle cells (SMCs) of 25‐week‐old WT, 25‐week‐old CerS2‐null, and 100‐week‐old mice. The IBTx‐sensitive currents (c) were measured as the K_C__a_1.1 currents. (D) *I*–*V* relationships for the gastric SMCs (left panel; *n *= 7) and normalized *I*–*V* curves from left panel (right panel). The amplitudes of the currents were normalized to the current measured at +80 mV. (E) Single‐channel currents obtained from an inside‐out patch and the amplitude histograms (*n *= 3–4; left panel). (F) Relationship between holding potential and current amplitude (*n *= 4–5; upper panel) or between [Ca^2+^]_i_ and NP_o_ (lower panel). *P < 0.05, **P < 0.01 vs. 25‐week‐old WT.

Altered SL composition due to aging or CerS2 ablation may affect ion channel expression on the cell surface and ion channel activity in GSM. We thus examined whether K_Ca_1.1 expression is altered in the GSM of aged WT or CerS2‐null mice. First, we compared K_Ca_1.1 levels in GSM from 25‐ and 100‐week‐old mice and found that the levels of α‐ and β‐subunits were significantly higher in the GSM of 100‐week‐old mice compared with that of 25‐week‐old mice (Fig. 2A). K_Ca_1.1 (α‐subunit) levels in GSM were shown to increase in an age‐dependent manner from 15‐ to 60‐week‐old mice (Fig. S6A, Supporting information). In addition, we demonstrated that K_Ca_1.1 (α‐subunit, both mRNA and protein) levels were significantly higher in the GSM of young (25 weeks old) CerS2‐null mice (Fig. 2B) and in cultured gastric SMCs from young CerS2‐null mice (Fig. S2, Supporting information) compared to WT mice of the same age. Additionally, β‐subunit levels were also increased in the GSM of young CerS2‐null mice (Fig. 2B).

Next, we examined whether whole‐cell K_Ca_1.1 currents are increased in gastric SMCs from aged WT and young CerS2‐null mice. K_Ca_1.1 currents were markedly increased, while the current–voltage (*I*–*V*) relationship remained unaffected, in aged WT and young CerS2‐null gastric SMCs (Fig. 2C,D). We then examined whether the single‐channel activity of K_Ca_1.1 is increased in the gastric SMCs of aged WT and young CerS2‐null mice (Fig. 2E,F). The conductance of K_Ca_1.1 channels in gastric SMCs of young WT, young CerS2‐null, or aged WT mice was 121.4 ± 11.1 pS, 128.5 ± 7.9 pS, and 129.3 ± 12.5 pS, respectively, with no significant difference between the three conductances (upper panel of Fig. 2F). In addition, the Ca^2+^ sensitivity of the K_Ca_1.1 channels in gastric SMCs was unchanged in young WT, young CerS2‐null, or aged WT mice because the Ca^2+^–*NP*_*O*_ relationship was not altered in the gastric SMCs from these mice (lower panel of Fig. 2F). These results indicate that the biophysical properties of the K_Ca_1.1 channels did not differ between young WT, young CerS2‐null, or aged WT mice. Thus, the increase in K_Ca_1.1 currents in the SMCs of aged WT and CerS2‐null mice might be caused by the simultaneous increase in levels of α‐ and β‐subunits on the cell membrane. The β‐subunit modifies biophysical properties (the Ca^2+^ and voltage sensitivity) of the pore‐forming α‐subunits (McManus *et al*., 1995). Because the biophysical properties of the K_Ca_1.1 channels were not changed, we did not examine the levels of β‐subunits thereafter.

These results suggest that K_Ca_1.1 expression on the cell membrane is increased in the gastric SMCs from aged WT and CerS2‐null mice and thereby increases K_Ca_1.1 currents without affecting the single‐channel activity of K_Ca_1.1.

### Altering SL composition induces K_Ca_1.1 upregulation

**Figure 3:**
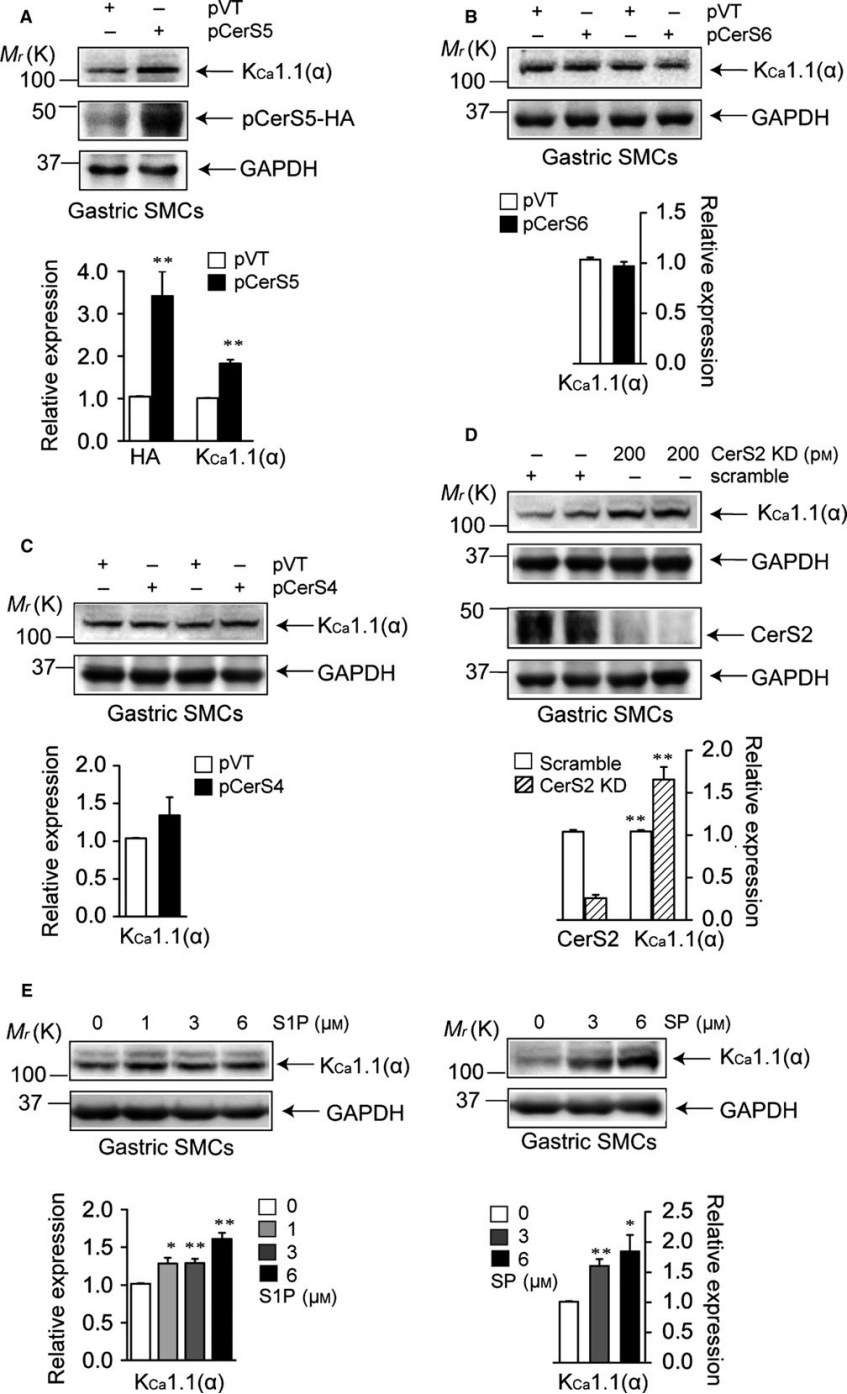
Roles of sphingolipids (SLs) on K_C__a_1.1 upregulation. (A–C) K_C__a_1.1 protein levels 24 h after transfection of CerS5 (A), CerS6 (B), or CerS4 (C) to WT gastric smooth muscle cells (SMCs). (D) K_C__a_1.1 and CerS2 protein levels 24 h after transfection of WT gastric SMCs with either scrambled siRNA or siRNA against CerS2. (E) K_C__a_1.1 protein levels 24 h after treatment of WT gastric SMCs with sphingosine 1‐phosphate (S1P) or SP. Blots are representative of 3–4 experiments. Results have been normalized to GAPDH levels. *P < 0.05, **P < 0.01 vs. gastric SMCs transfected with an empty vector (A–C), transfected with scrambled siRNA (D), or treated with vehicle (E).

As mRNA levels of CerS4, CerS5, and CerS6 were increased and CerS2 mRNA levels were decreased in aged WT and young CerS2‐null mice, we examined whether the upregulation of CerS4, CerS5, or CerS6, or the downregulation of CerS2, induces K_Ca_1.1 upregulation in aged WT and young CerS2‐null mice. CerS5, CerS6, or CerS4 was transfected into the gastric SMCs of WT mice. CerS5 transfection did not affect the levels of other CerS such as CerS2, CerS4, or CerS6 (Fig. S3A, Supporting information), and neither did CerS4 nor CerS6 transfection (data not shown). CerS5 transfection significantly upregulated K_Ca_1.1 (Fig. 3A). However, CerS6 (Fig. 3B) or CerS4 transfection (Fig. 3C) did not cause a change in K_Ca_1.1 levels. Next, we examined the effect of CerS2 knockdown on K_Ca_1.1 levels using siRNA against CerS2. CerS2 knockdown upregulated K_Ca_1.1 in the gastric SMCs of WT mice (Fig. 3D), without affecting levels of other CerS in gastric SMCs (Fig. S3C). LC‐MS/MS analysis of ceramide levels exhibited that the levels of C16‐ and C18‐ceramides were increased and those of C22, C24:1, and C24 ceramides were decreased in CerS5‐transfected (Fig. S3B) or CerS2 knocked down (Fig. S3D) gastric SMCs. In addition, we examined whether SP or S1P affects K_Ca_1.1 levels because SP and S1P were increased in aged WT and young CerS2‐null mice. Exogenously added SP or S1P upregulated K_Ca_1.1 in primary WT gastric SMCs in a concentration‐dependent manner (Fig. 3E). These results suggest that altering SL composition increases K_Ca_1.1 expression in gastric SMCs.

### K_Ca_1.1 upregulation reduces the contractility of gastric smooth muscle

**Figure 4:**
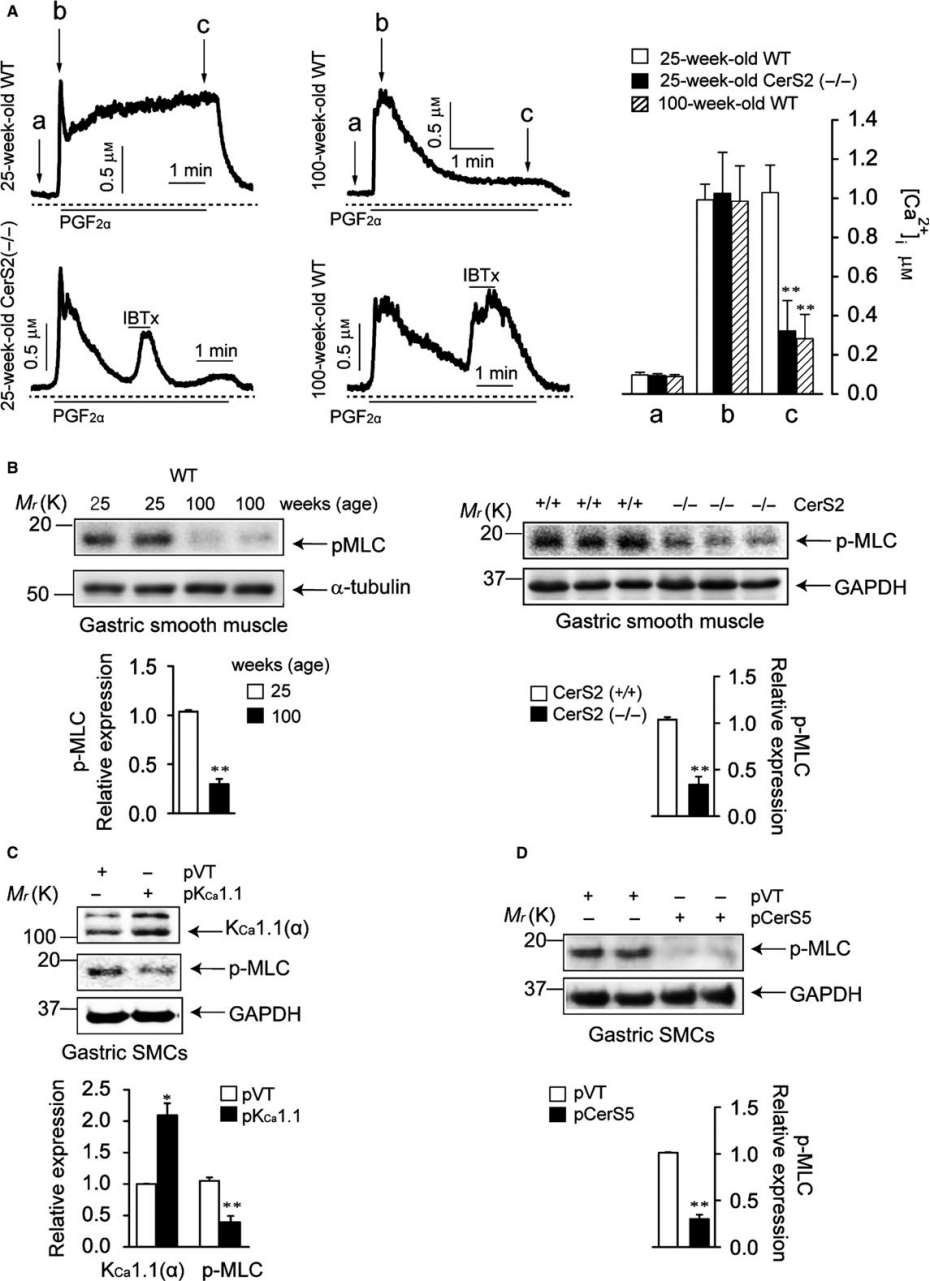
K_C__a_1.1 upregulation inhibits an increase in intracellular Ca^2+^ concentration ([Ca^2+^]_i_) and myosin light chain (MLC) phosphorylation. (A) [Ca^2+^]_i_ was measured in the gastric smooth muscle cells (SMCs) of young WT, aged WT, and young CerS2‐null mice. Gastric SMCs were stimulated with prostaglandin F_2α_ (PGF_2α_), and iberiotoxin (IBTx) was used to block K_C__a_1.1. Summary data are shown in the left panel (*n* = 4). (B) Protein levels of phosphorylated MLC (p‐MLC) were examined in gastric smooth muscle of aged WT, young CerS2‐null, and age‐matched WT mice. (C,D) K_C__a_1.1 (α‐subunits) and p‐MLC levels were measured 24 h after K_C__a_1.1 (C) or CerS5 (D) transfection of WT gastric SMCs. (B–D) Blots are representative of 3–4 experiments. Results are normalized to GAPDH or α‐tubulin levels. *P < 0.05, **P < 0.01 vs. young WT (A,B) or WT gastric SMCs transfected with empty vector (C,D).

As K_Ca_1.1 activation inhibits Ca^2+^ influx through VOCCs, an increased expression of K_Ca_1.1 might impair intracellular Ca^2+^ mobilization in SMCs. We thus measured [Ca^2+^]_i_ in the gastric SMCs of 100‐week‐old WT, 25‐week‐old CerS2‐null, and 25‐week‐old WT mice (Fig. 4A). Prostaglandin F_2α_ (PGF_2α_, 10 μm) increased [Ca^2+^]_i_ in these cells. In young WT gastric SMCs, the increased [Ca^2+^]_i_ was sustained in the presence of PGF_2α_. In contrast, aged WT and young CerS2‐null gastric SMCs showed a rapid decline in the increased [Ca^2+^]_i_ despite the presence of PGF_2α_, and the decline in [Ca^2+^]_i_ was inhibited by the selective K_Ca_1.1 channel blocker iberiotoxin (IBTx, 300 nm). These observations suggest that K_Ca_1.1 upregulation induces dysregulation of Ca^2+^ signals by exerting a negative feedback on Ca^2+^ signals in gastric SMCs. The dysregulation of Ca^2+^ signals was further confirmed by measuring the levels of p‐MLC in the GSM of 100‐week‐old WT, 25‐week‐old CerS2‐null, and 25‐week‐old WT mice (Fig. 4B), as Ca^2+^ plays a critical role in MLC phosphorylation (Kamm & Stull, 1989). Levels of p‐MLC were significantly smaller in aged WT and young CerS2‐null GSM. In addition, p‐MLC levels were significantly decreased by K_Ca_1.1 transfection (Fig. 4C) or by CerS5 transfection (Fig. 4D) in WT gastric SMCs, suggesting that K_Ca_1.1 upregulation by altering SL composition reduces p‐MLC levels. The inverse relationship between K_Ca_1.1 and p‐MLC expression was further confirmed in the GSM of 25‐week‐old CerS2‐null and age‐matched WT mice by immunohistochemical analysis (Fig. S4, Supporting information). Compared to the GSM in WT mice, green fluorescence of K_Ca_1.1 was increased and red fluorescence of p‐MLC was decreased in that of CerS2‐null mice, suggesting that CerS2 ablation induces K_Ca_1.1 upregulation and p‐MLC downregulation simultaneously in GSM.

**Figure 5:**
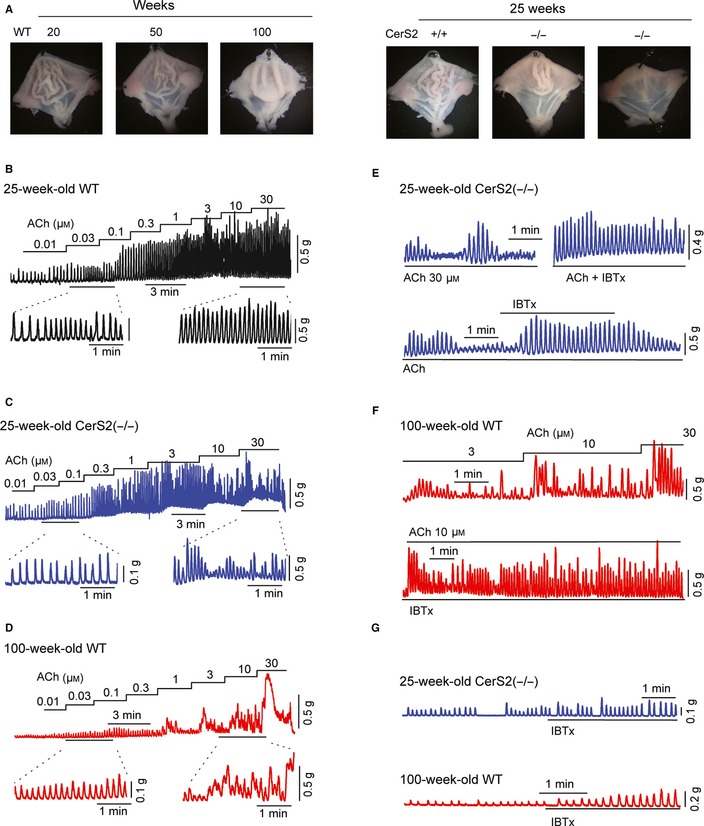
Contractile dysfunction is developed in gastric smooth muscle (GSM) of aged WT or young CerS2‐null mice. (A) Mucosal folds in the inner surface of the stomach were compared between WT and CerS2‐null mice. (B–D) ACh‐induced contraction of GSM of young WT (B), young CerS2‐null (C), and aged WT (D) mice. (E,F) The specific K_C__a_1.1 blocker, iberiotoxin (IBTx), recovered ACh‐induced contractile dysfunction of GSM from young CerS2‐null (E) and aged WT (F) mice. (G) Spontaneous contractions of GSM from CerS2‐null (upper panel) and aged WT (lower panel) mice.

The dysregulation of Ca^2+^ signals and decreased MLC phosphorylation suggests that contractile dysfunction develops in the GSM of aged WT and young CerS2‐null mice. When the inner surface of the stomach was compared between mice, the number of mucosal folds was remarkably decreased in an age‐associated manner or in CerS2‐null mice (Fig. 5A). Mucosal folds may be caused by shortening of the underlying GSM, suggesting that a decrease in the number of mucosal folds is caused by a decrease in contractility. We therefore compared contractility of GSM between young WT (Fig. 5B), young CerS2‐null (Fig. 5C), and aged WT (Fig. 5D) mice. When the smooth muscle was exposed to ACh, the frequency and magnitude of phasic contraction of GSM were increased in a concentration‐dependent manner. The GSM of young WT mice maintained regular rhythmic contraction in the presence of ACh from concentrations of 0.01 to 30 μm, whereas that of young CerS2‐null and aged WT mice did not. When the GSM from aged WT or CerS2‐null mice was exposed to low concentrations of ACh, phasic contractions were increased in the frequency and magnitude in a concentration‐dependent manner, and well maintained in the presence of ACh, the response of which was similar to that of young WT mice. In contrast, when the smooth muscle was exposed to high concentrations of ACh, the frequency and magnitude of each contraction became irregular. The magnitude of the phasic contraction was not maintained and markedly decreased despite the presence of ACh (Fig. 5C,D). These results suggest that contractile dysfunction of GSM is induced by high concentrations of ACh in aged WT and CerS2‐null mice. The contractile dysfunction of GSM in CerS2‐null (Fig. 5E) and aged WT (Fig. 5F) mice was recovered to regular phasic contraction by IBTx (300 nm) treatment. In addition, the frequency of spontaneous contraction was significantly decreased in aged WT (5.2 ± 0.3 min^−1^, *n* = 9) and young CerS2‐null (5.0 ± 0.2 min^−1^, *n* = 10) mice compared to young WT mice (6.9 ± 0.2 min^−1^, *n* = 13). IBTx treatment significantly increased the frequency in aged WT (5.7 ± 0.2 min^−1^, *n* = 9) and young CerS2‐null (5.6 ± 0.3 min^−1^, *n* = 10) mice (Fig. 5G). The magnitude of spontaneous contraction was also increased by IBTx treatment. We then used tetrodotoxin to exclude neural influences (Fig. S5, Supporting information). When GSM was pretreated with the Na^+^ channel blocker tetrodotoxin (1 μm), contractile dysfunction was induced by high concentrations of ACh in the GSM of young CerS2‐null and aged WT mice, and IBTx treatment recovered the contractile dysfunction to regular phasic contractions, suggesting that the contractile dysfunction by high concentrations of ACh occurs independently of neural influences.

These results suggest that K_Ca_1.1 upregulation due to aging or CerS2 ablation induces contractile dysfunction in GSM.

### K_Ca_1.1 upregulation is induced via the PI3K/PKC_ζ_/JNK/NF‐κB‐mediated pathway

**Figure 6:**
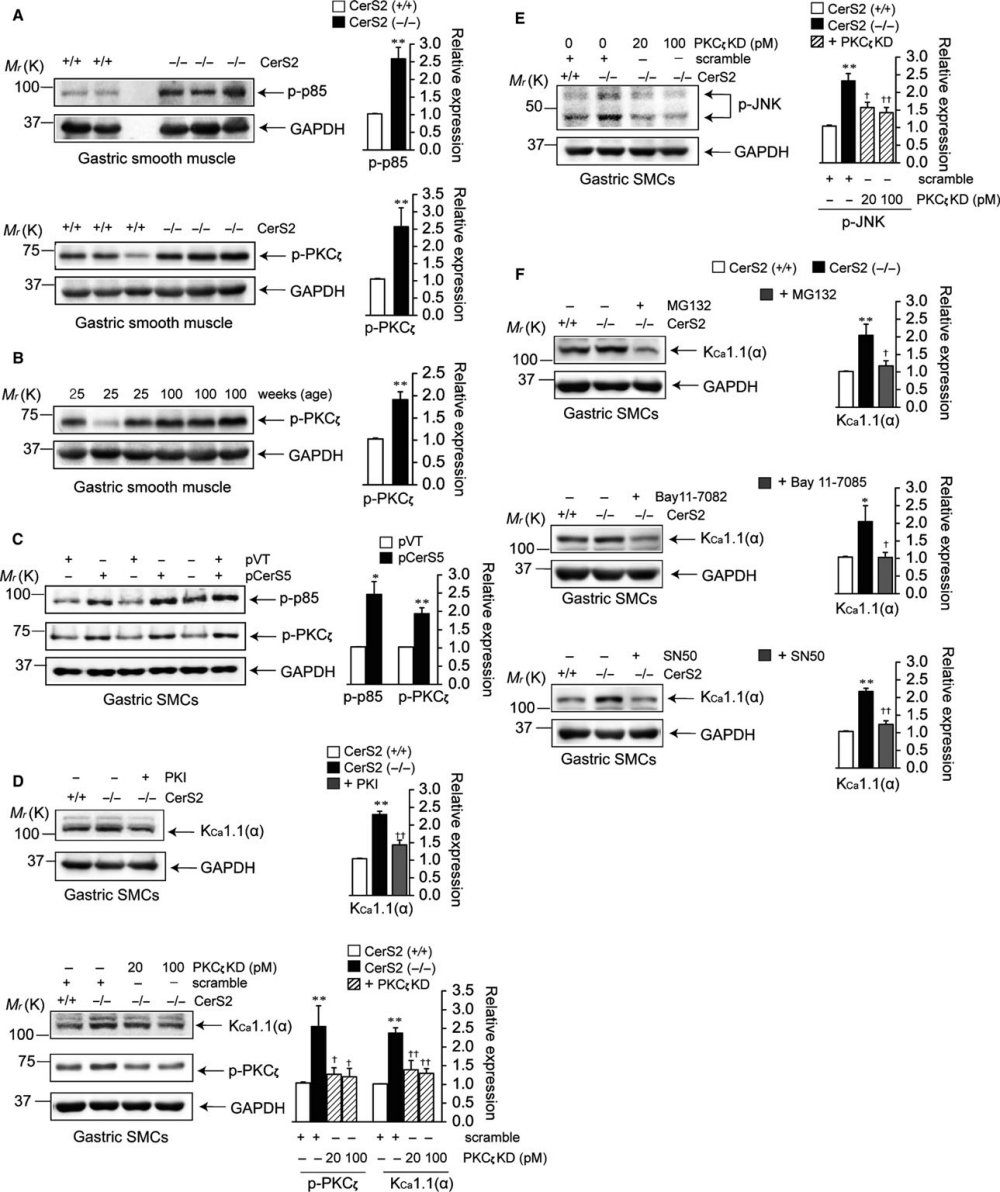
The PI3K/PKC_ζ_/JNK/NF‐κB pathway mediates K_C__a_1.1 upregulation due to aging or ceramide synthases 2 (CerS2) ablation. Protein levels of phosphorylated p85 (p‐p85), phosphorylated PKC_ζ_ (p‐PKC_ζ_), K_C__a_1.1, and phosphorylated JNK (p‐JNK) were examined in gastric smooth muscle (GSM) or primary cultured gastric smooth muscle cells (SMCs). (A) Levels of p‐p85 or p‐PKC_ζ_ in GSM of young CerS2‐null and age‐matched WT mice. (B) p‐PKC_ζ_ levels in GSM of WT mice at different ages. (C) p‐PKC_ζ_ levels in WT gastric SMCs transfected with CerS5 for 24 h. (D) K_C__a_1.1 levels in CerS2‐null gastric SMCs treated with 10 μm PKC_ζ_ pseudosubstrate inhibitor (PKI) for 24 h (upper panels), or in CerS2‐null gastric SMCs transfected with scrambled siRNA or siRNA against PKC_ζ_ for 24 h (lower panels). (E) p‐JNK levels in CerS2‐null gastric SMCs transfected with scrambled siRNA or siRNA against PKC_ζ_ for 24 h. (F) K_C__a_1.1 levels in CerS2‐null gastric SMCs treated with the NF‐κB blockers, MG132, Bay11‐7082, or SN50, for 24 h. The blots are representative of 3–4 experiments. Results were normalized to GAPDH or α‐tubulin levels. *P < 0.05, **P < 0.01 vs. WT GSM or SMCs treated with scrambled siRNA or empty vector. ^*†*^ *P *< 0.05, ^*††*^ *P *< 0.01 vs. CerS2‐null gastric SMCs treated with scrambled siRNA or vehicle.

Ceramides mediate several intracellular signals via second messengers, including kinases, phosphatases, proteases, or transcription factors (Levade *et al*., 2001). We hypothesized that the altered SL profile might preferentially affect the PI3K pathway (Hirsch *et al*., 2007; Silva *et al*., 2012). It was shown that levels of phosphorylated p85 (p‐p85) were higher in the GSM of young (25 weeks old) CerS2‐null mice (upper panel of Fig. 6A). Several studies have reported that PI3K is directly involved in the activation of PKC_ζ_ (Le Good *et al*., 1998) which can be activated by ceramides (Wang *et al*., 1999). Levels of phosphorylated PKC_ζ_ (p‐PKC_ζ_) were upregulated in the GSM of young CerS2‐null (lower panel of Fig. 6A) and aged WT (Fig. 6B) mice. In addition, levels of p‐p85 and p‐PKC_ζ_ were increased by CerS5 transfection in WT gastric SMCs (Fig. 6C). Involvement of PKC_ζ_ in the regulation of K_Ca_1.1 expression by CerS2 ablation was further examined using the PKC_ζ_ pseudosubstrate inhibitor (PKI) or siRNA against PKC_ζ_ in primary gastric SMCs (Fig. 6D). K_Ca_1.1 upregulation was reduced by treatment with PKI or siRNA against PKC_ζ_ in CerS2‐null gastric SMCs, suggesting that PKC_ζ_ is involved in K_Ca_1.1 upregulation by CerS2 ablation. In addition, the levels of phosphorylated JNK (p‐JNK) were increased in CerS2‐null gastric SMCs, which was reversed by PKC_ζ_ knockdown in CerS2‐null gastric SMCs (Fig. 6E). Finally, the involvement of NF‐κB in the transcriptional regulation of K_Ca_1.1 was directly tested using the NF‐κB inhibitors, MG132 (2.5 μm), Bay 11‐7082 (5 μm), or SN50 (30 μg mL^−1^), in CerS2‐null gastric SMCs. K_Ca_1.1 upregulation was reversed by these NF‐κB inhibitors in CerS2‐null gastric SMCs (Fig. 6F). Our results suggest that an altered SL profile by CerS2 downregulation and/or CerS5 upregulation induces K_Ca_1.1 upregulation in gastric SMCs through the PI3K/PKC_ζ_/JNK/NF‐κB pathways.

## Discussion

The results of our study show that altered SL composition due to aging or CerS2 ablation upregulates K_Ca_1.1 via the PI3K/PKC_ζ_/JNK/NF‐κB‐mediated pathway in GSM. K_Ca_1.1 upregulation inhibits Ca^2+^ influx and MLC phosphorylation and thereby reduces the contractility of GSM to evoke contractile dysfunction. In contrast, alteration of the SL acyl chain length or composition did not affect the single‐channel activity of K_Ca_1.1 in gastric SMCs. These findings represent the first evidence for the mechanism of the contractile dysfunction of GSM observed in older individuals, which may be implicated in age‐associated gastrointestinal motility disorders.

When SMCs are stimulated by agonists, [Ca^2+^]_i_ is increased by Ca^2+^ being released from intracellular Ca^2+^ stores and Ca^2+^ influx through VOCCs. The initial peak is evoked by the release, and Ca^2+^ influx through VOCCs maintains elevated [Ca^2+^]_i_. In contrast, K_Ca_1.1 exerts a negative feedback on Ca^2+^ influx by closing VOCCs. Therefore, K_Ca_1.1 upregulation can impair Ca^2+^ mobilization via VOCC inhibition and thereby reduce the contractility of smooth muscle, as proven by decreased p‐MLC by K_Ca_1.1 transfection. Thus, K_Ca_1.1 expression on the cell membrane plays an important role in myogenic regulation of gastrointestinal motility. The negative contribution of K_Ca_1.1 to the contractility of smooth muscle seems to be increased with age, as the levels of K_Ca_1.1 were increased with advancing age. Consistent with our results, age‐dependent K_Ca_1.1 upregulation was reported in mouse pancreatic acinar cells (Oshiro *et al*., 2005) and in rat skeletal muscle fibers (Tricarico *et al*., 1997). Age‐dependent K_Ca_1.1 upregulation seems to cause contractile dysfunction with aging. Supporting this suggestion, we observed that K_Ca_1.1 inhibition recovers contractile dysfunction of GSM in aged WT mice. K_Ca_1.1 inhibition increased the reduced frequency and amplitude of spontaneous contraction and recovered ACh‐induced phasic contraction from irregular to regular rhythm in the GSM of aged WT and young CerS2‐null mice. Together, with the degeneration of ICC and the enteric nervous system with advancing age, contractile dysfunction of smooth muscle by K_Ca_1.1 upregulation might contribute to the development of age‐associated gastrointestinal motility disorder.

It was shown that SP or S1P upregulated K_Ca_1.1; however, the mechanism underlying K_Ca_1.1 upregulation by SP or S1P is still unknown. K_Ca_1.1 upregulation by CerS2 knockdown suggests that CerS2 negatively contributes to K_Ca_1.1 expression, and an inhibitory effect of S1P on CerS2 activity (Laviad *et al*., 2008) suggests that S1P upregulates K_Ca_1.1 by inhibiting CerS2. In addition, K_Ca_1.1 upregulation by CerS5 transfection suggests that CerS5 positively contributes to K_Ca_1.1 expression. As CerS5 synthesizes the C16‐ceramide, K_Ca_1.1 upregulation might, at least in part, be caused by an increase in C16‐ceramide levels. However, CerS2 knockdown or CerS5 transfection induced not only a decrease in C22 and C24 ceramide content, but also an increase in C16‐ and C18‐ceramide content. We therefore suggest that K_Ca_1.1 upregulation in the gastric SMCs of aged WT and young CerS2‐null mice is caused by an altered SL profile, that is, increased C16‐SL, decreased C22‐C24‐SL, or increased long‐chain bases such as SP or sphinganine.

Although CerS4 was upregulated in the gastric SMCs of CerS2‐null mice, the contribution of the altered SL profile by CerS4 to K_Ca_1.1 upregulation seems to be negligible because CerS4 transfection increased K_Ca_1.1 levels to a very small extent. In addition, the contribution of CerS6 to K_Ca_1.1 upregulation is also negligible because CerS6 transfection did not affect K_Ca_1.1 levels. This is unexpected, because CerS6 also synthesizes the C16‐ceramide, and indicates that the C16‐ceramide that is formed by CerS5 might be functionally different from the C16‐ceramide formed by CerS6.

PI3K activates PKC_ζ_ (Le Good *et al*., 1998) and PKC_ζ_ inhibition using a specific blocker or siRNA against PKC_ζ_ reversed the increase in K_Ca_1.1 levels in the gastric SMCs of CerS2‐null mice, suggesting that PI3K activation plays a critical role in K_Ca_1.1 upregulation. PI3K activation by phosphorylation might be caused by a change in lipid rafts, which are associated with several signal transduction molecules including Src family kinases and small G proteins (Yoshizaki *et al*., 2008) and act as important regulators of PI3K (Gao *et al*., 2011). In addition, K_Ca_1.1 is localized to caveolae (Brainard *et al*., 2005), a subset of lipid rafts, and has a caveolin‐binding motif (Suzuki *et al*., 2013). Thus, a direct interaction between K_Ca_1.1 and caveolin regulates K_Ca_1.1 expression at the cell surface (Suzuki *et al*., 2013). As alteration of lipid rafts was suggested in CerS2‐null mice (Silva *et al*., 2012; Park *et al*., 2013) and SLs are important structural component of membranes, altering the SL composition might cause changes in lipid rafts. In addition, PI3K activation results in JNK activation (Kobayashi *et al*., 1997; Wang *et al*., 1999), and PKC_ζ_ is activated by ceramides, resulting in coactivation of NF‐κB and JNK (Wang *et al*., 1999). Collectively, these findings suggest that the PI3K/PKC_ζ_/JNK/NF‐κB pathway plays a key role in K_Ca_1.1 upregulation by altered SL composition with aging or by CerS2 ablation.

The contribution of ion channels to cellular function is determined by the single‐channel activity of ion channels and the number of channels expressed on cell membranes. As the single‐channel activity of K_Ca_1.1 was not affected with aging or by CerS2 ablation, the increase in the whole‐cell K_Ca_1.1 currents in the gastric SMCs of aged WT and young CerS2‐null mice might be due to the increased expression of K_Ca_1.1 on the cell surface. Thus, the alternation in acyl chain length or composition of SLs affects K_Ca_1.1 expression on the cell surface, without affecting the single‐channel activity of K_Ca_1.1. The expression of membrane proteins, including ion channels in the plasma membrane, is determined by a balance between synthesis/forward trafficking from the endoplasmic reticulum, endocytosis, and recycling/degradation (Conn & Ulloa‐Aguirre, 2010; Simms & Zamponi, 2012). Recent studies demonstrate that various K^+^ channel proteins undergo endocytosis and degradation via PKC activation or by glycosphingolipids. PKC activation significantly decreased the expression of ATP‐sensitive K^+^ channels on the cell membrane by reducing channel recycling and diverting the channel to lysosomal degradation (Manna *et al*., 2010). The neutral glycosphingolipid, globotriaosylceramide, inhibited K_Ca_3.1 synthesis (Park *et al*., 2011) and induced lysosomal degradation of K_Ca_3.1 by facilitating channel protein internalization from the plasma membrane in endothelial cells (Choi *et al*., 2014), when globotriaosylceramide was excessively accumulated in endothelial cells. In addition, sphingomyelin, a major SL in mammalian cell membranes, is enriched in the plasma membrane and in the endocytic recycling compartment and may be involved in the regulation of endocytosis (Slotte, 2013). Therefore, altering SL composition may affect K_Ca_1.1 expression on the cell membrane by regulating synthesis/forward trafficking from the endoplasmic reticulum, endocytosis, and the recycling/degradation of K_Ca_1.1. Further studies are required to evaluate whether altering SL composition affects the regulation of K_Ca_1.1 and thereby increases K_Ca_1.1 expression on cell membrane.

The CerS2‐null mice share similarities with the pathophysiological changes observed in patients or mouse models of diabetes mellitus, as insulin resistance and glucose intolerance were observed in CerS2‐null mice (Park *et al*., 2013). SLs and ceramides affect insulin signaling by phosphorylation of protein kinase B or insulin receptors (Stratford *et al*., 2004; Langeveld & Aerts, 2009). In addition, ceramides generated by CerS5 were responsible for lipotoxic cardiomyopathy and hypertrophy in mouse models of diabetic cardiomyopathy (Russo *et al*., 2012). Decreased contractility of GSM is frequently encountered in patients with diabetes mellitus. Therefore, further studies are required to evaluate whether K_Ca_1.1 upregulation occurs, which thereby decreases the contractility of GSM in patients with diabetes mellitus.

The CerS2‐null mice recapitulate important characteristics of the aged and therefore allow us to investigate the role of SLs in the function of GSM, including its role in K_Ca_1.1 expression. Characterization of gastric SMCs from young CerS2‐null mice showed an altered SL profile, K_Ca_1.1 upregulation, impaired Ca^2+^ signaling, and reduced p‐MLC, which mimic the alterations found in the aged mice. In addition, the altered SL composition induced similar contractile dysfunction of GSM by upregulating K_Ca_1.1 in both mice, and the recovery of contractile dysfunction by K_Ca_1.1 inhibition was similar in both mice. Furthermore, levels of the cyclin‐dependent kinase inhibitor p21 (p21^*CIP1*^), which is expressed in senescent cells, were markedly increased in the GSM or in primary gastric SMCs from aged WT and young CerS2‐null mice (Fig. S6). Thus, we suggest that the CerS2‐null mouse model is valuable for understanding the pathogenesis of aging‐related motility disorders as well as for studies toward the design of therapeutic approaches.

In conclusion, our data suggest that aging or CerS2 ablation alters SL acyl chain length and composition, which induces K_Ca_1.1 upregulation via the PI3K/PKC/JNK/NF‐κB signaling pathway and thereby causes contractile dysfunction of GSM. To the best of our knowledge, this is the first study to demonstrate the mechanisms underlying age‐dependent contractile dysfunction of smooth muscle and an age‐dependent increase in endogenous K_Ca_1.1 expression in native gastric SMCs. In addition, we suggest that CerS2‐null mice may serve as a model for contractile dysfunction that is developed in the aged. Therefore, CerS2‐null mice may provide an additional resource for understanding the underlying mechanisms responsible for changes in ion channel expression by altered SL composition.

## Experimental procedures

For description of contraction measurement on gastric muscle strips, electrophysiological recording, Ca^2+^ measurement, PCR, Western blotting, immunostaining, and LC‐MS/MS analysis of SLs, please refer to the Appendix S1 (Supporting information).

### Animals

The CerS2 null mice were generated as described (Levy & Futerman, 2010). The investigation was approved by the local ethics committee, the Institutional Review Board of the Ewha Womans University, and was in accordance with the Declaration of Helsinki, the Animal Care Guidelines of the Ewha Womans University, Medical School, and the National Institutes of Health Guide for the Care and Use of Laboratory Animals.

### Cell isolation and culture

The mucosal layer of stomach was separated from the muscle layer in Ca^2+^‐free physiological salt solution, and gastric SMCs were isolated from GSM segments. The GSM layer was cut into small segments (2 × 3 mm), which were incubated for 15–25 min at 37 °C in digestion medium containing 0.1% collagenase (Wako Pure Chemicals, Osaka, Japan), 0.05% DTT, 0.1% trypsin inhibitor, and 0.2% BSA. After digestion, gastric SMCs were harvested by gentle agitation with a wide‐bore glass pipette.

### Transfection

A K_Ca_1.1 plasmid was provided by Dr. Chul‐Seung Park of Gwangju Institute of Science and Technology (Korea). Gastric SMCs were transiently transfected for 24 h with each plasmid using JET‐PEI (Polyplus‐Transfection Inc, New York, NY, USA) or Effectene (Qiagen Inc, Valencia, CA, USA) according to the procedure suggested by the manufacturer. To knockdown CerS2 in gastric SMCs, scrambled siRNA or siRNA against CerS2 was purchased from Santa Cruz Biotechnology (Santa Cruz, CA, USA). Gastric SMCs were transiently transfected for 24 h with the siRNAs using siRNA transfection reagent (Santa Cruz Biotechnology).

### Chemicals

Ceramides (acyl chain lengths of C14, C16, C18, C22, C24, and C24:1), C17‐ceramide (d17:1/C18:0), sphinganine, SP, S1P and C_17_‐SP, C_17_‐S1P as an internal standard were obtained from Avanti Polar Lipids (Alabaster, AL, USA). Organic solvents for SL extraction and HPLC analysis were purchased from Merck (Darmstadt, Germany). ACh, Bay11‐7082, IBTx, MG132, PGF_2α_, PKI, SN50, and tetrodotoxin were purchased from Sigma‐Aldrich (St Louis, MO, USA). The primary antibodies used in this study were anti‐Tyr‐458‐p‐p85 (Cell Signaling Technology, Boston, MA, USA), anti‐Thr‐560‐p‐PKC_ζ_ (Abcam, Cambridge, MA, USA), anti‐Thr‐183/Tyr‐185‐p‐JNK (Cell Signaling Technology), anti‐Ser‐19‐p‐MLC (Cell Signaling Technology), anti‐K_Ca_1.1 (α‐subunit; Novus Biologicals, Littleton, CO, USA), anti‐K_Ca_1.1 (β‐subunit; Abcam), anti‐CerS2 (Sigma‐Aldrich), anti‐CerS5 (Santa Cruz), and anti‐GAPDH (EMD Millipore, Darmstadt, Germany).

### Statistical analysis

Data are means ± SEM for 3–7 independent experiments. To examine the statistical significance between groups, one‐way anova or two‐tailed Student's *t*‐test was used. A *P* value of 0.05 or lower was considered statistically significant.

## Author contributions

Shinkyu Choi performed study concept and design, acquisition of data, analysis and interpretation of data, drafting of the manuscript, critical revision of the manuscript for intellectual content; Tae Hun Kim and Seikwan Oh performed analysis and interpretation of data, technical support; Jee Aee Kim, Hae‐yan Li, and Kyong‐Oh Shin performed acquisition, analysis, and interpretation of data; Yong‐Moon Lee performed acquisition of data, analysis and interpretation of data, technical support, critical revision of the manuscript for intellectual content; Yael Pewzner‐Jung supplied the CerS2 null mice and critical revision of the manuscript for intellectual content; Anthony H. Futerman supplied the CerS2 null mice and critical revision of the manuscript for intellectual content, material support, obtaining funding; Suk Hyo Suh performed study concept and design, analysis and interpretation of data, drafting of the manuscript, critical revision of the manuscript for intellectual content, obtained funding.

## Funding

This research was supported by Basic Science Research Program through the Nation Research Foundation of Korea funded by the Ministry of Education, Science and Technology (R01‐2010‐000‐10466‐0, NRF‐2013R1A1A2010851, NRF‐2013R1A1A2064543), by the National Research Foundation of Korea Grant funded by the Korean Government (NRF‐2010‐220‐E00001), and by the Israel Science Foundation (0888/11). A.H. Futerman is The Joseph Meyerhoff Professor of Biochemistry at the Weizmann Institute. of Science.

## Conflict of interest

None declared.

## Supporting information


**Appendix S1.** Supplementary Materials and methods.
**Fig. S1** Changes in levels of CerS and SLs in gastric SMCs by CerS2 ablation.
**Fig. S2** K_Ca_1.1 levels in primary cultured gastric SMCs from CerS2‐null mice.
**Fig. S3** Changes in levels of ceramides with various acyl chain lengths by CerS5 transfection or CerS2 knock‐down.
**Fig. S4** Inverse relationship between expression levels of K_Ca_1.1 and p‐MLC.
**Fig. S5** Tetrodotoxin did not prevent contractile dysfunction of aged WT or young CerS2‐null gastric smooth muscle.
**Fig. S6** p21^*CIP1*^ upregulation in gastric smooth muscle from aged WT and CerS2‐null mice.
