# Supplementary material for: Altering sphingolipid composition with aging induces contractile dysfunction of gastric smooth muscle via KC a1.1 upregulation
Source: Aging Cell. 2015 Aug 20;14(6):982–94. doi: 10.1111/acel.12388 (PMC4693452; doi:10.1111/acel.12388)

**Supporting Information**

**Supplementary Materials and Methods**

## Contraction measurement on isolated gastric muscle strips

The gastric smooth muscle layer without the mucosal layer was cut into small segments (3×8 mm) in the direction of circular muscle. The mechanical responses of the segments were recorded using a home-made myograph. In this myograph, one end of the tissue was tied tightly to a fixed holder and the other end was connected to a mechano-transducer (FT03, Natus Neurology Incorporated, Middleton, WI) mounted on a three dimensional manipulator. The muscle chamber was perfused at a flow rate of 2.5 ml/min with oxygenated (95% O_2_/5% CO_2_) Krebs/Ringer bicarbonate solution with a peristaltic pump. The composition (in mM) of the Krebs solution was NaCl 118.3, KCl 4.7, MgCl_2_ 1.2, KH_2_PO_4_ 1.22, CaCl_2_ 2.5, NaHCO_3_ 25.0, glucose 11.1, pH 7.4.

**Electrophysiological recordings**

Whole cell currents were measured using ruptured patches. The currents were monitored in the voltage-clamp mode with an EPC-9 (HEKA Elektronik, Lambrecht, Germany), sampling rate 1 ms, 8-pole Bessel filter 2.9 kHz. We applied a voltage steps from −80 mV to +80 mV (increment 20 mV, 1 second duration) with a 5 second interval from a holding potential of -60 mV. Inside-out voltage clamps were performed using glass electrodes of tip resistance of 8-10 MΩ. Data were filtered at 1 KHz and stored in a computer for analysis using standard software (Axoscope 9.0, Axon Instruments, Foster City, CA, USA).

The standard external solution contained (in mM): 150 NaCl, 6 KCl, 1.5 CaCl_2,_ 1 MgCl_2_, 10 HEPES, and 10 glucose; the pH was adjusted to 7.4 with NaOH. The pipette solution used for whole-cell recording contained (in mM): 40 KCl, 100 K-aspartate, 2 MgCl_2_, 4 Na_2_ATP, and 10 HEPES; the pH was adjusted to 7.2 with KOH. The K_Ca_1.1 currents were activated by loading cells with Ca^2+^ via a patch pipette. The K_Ca_1.1 currents were normalized to cell capacitance, and the iberiotoxin (IBTx, 300 nM)-sensitive currents were measured as the K_Ca_1.1 currents. The experiment was performed at room temperature (20–22°C).

In the inside-out mode, the external solution contained (in mM): 150 KCl, 0.5 MgCl_2_, 10 HEPES, 5 EGTA, 0~10 x10^-3^ free Ca^2+^, pH 7.2; and the pipette solution contained (in mM): 150 NaCl, 6 KCl, 1 MgCl_2_, 10 HEPES, 10 glucose, 5 EGTA, pH 7.4 with NaOH.

For buffering free Ca^2+^, an appropriate amount of Ca^2+^ (calculated by the CaBuf, software, G. Droogmans, Leuven, ftp://ftp.cc.kuleuven.ac.be/pub/droogmans/cabuf.zip) was added in the presence of 5 mM EGTA.

**Ca^2+^ measurements**

Cells were loaded with Fura-2 AM and the intracellular Ca^2+^ concentration was measured in isolated single cells using a microfluorometer consisting of an inverted microscope (DM IRB, Leica Microsystems GmbH, Wetzlar, Germany) and a PTI filter scan power illuminator system (Photon Technology International, Edison, NJ). Fura-2AM (2 μM) was added to the bath, and the cells were incubated for 25 min at 37°C. The cells were illuminated alternatively at wavelengths of 340 and 380 nm through a chopper wheel (at a frequency of 50 Hz). Fluorescence was measured at 510 nm, and autofluorescence was subtracted. The free Ca^2+^ concentration was calculated from the ratio of the fluorescence signals emitted at each excitation wavelength. The calibration procedure was identical to that described previously ([Nilius *et al.*, 1993](#_ENREF_1)).

**PCR**

RNA was isolated from the cells using the RNeasy Mini Kit (Qiagen, Valencia, CA), and then reverse transcribed using a High Capacity cDNA Archive Kit (Applied Biosystems, Foster City, CA). PCR was performed on an ABI 7000 sequence detection system (Applied Biosystems) using SYBR Green PCR Master Mix (Applied Biosystems) or PCR Thermal Cycler (BioRad, Hercules, CA). The following primers were used: CerS1 (5′-AGT CTG TGC CTG ACA TTC CG-3′ [sense] and 5′-GCC ATT CCT CAG TGG CTT CT-3′ [antisense]), CerS2 (5′- GCT GGA GAT TCA CGT ATT AC-3′ [sense] and 5′- GAA CAC AAT GAA GAG GTT GT-3′ [antisense]), CerS3 (5′- GAG CGC CAG GTT GAA AGA TG-3′ [sense] and 5′- TAC TGG GAC GGC AGC AAA G-3′ [antisense]), CerS4 (5′- CCG TGG GAC TGA TAG GCT TC-3′ [sense] and 5′- CGT GTA GAT GAC CTG GGT GG-3′ [antisense]), CerS5 (5′- CAA CTG GAC TGG AGT GTT CG-3′ [sense] and 5′- TCT CGA GAG TGG CTG ATA CG-3′ [antisense]), CerS6 (5′- CTG GAC TGG GAT GTT CGG AG-3′ [sense] and 5′- CAG CTG TGA GTG GCT GGT AA-3′ [antisense]), and GAPDH (5′- CTC CCA CTC TTC CAC CTT CG-3′ [sense] and 5′- TAG GGC CTC TCT TGC TCA GT-3′ [antisense]).

**Western Blotting**

Cell or tissue lysate samples were used. Total protein was measured using the bicinchoninic acid assay (Pierce Biotechnology). Protein was separated by SDS-PAGE on 7.5–12% gels and transferred to nitrocellulose membranes. The membrane was blocked using 5% BSA in Tris-buffered saline containing 0.1% Tween-20 (TBST) at room temperature for 1 hour. The primary antibody was diluted in TBST containing 5% BSA and incubated with the membrane overnight at 4°C. After washing 4 times for 5 min, membranes were incubated with the secondary antibody in TBST containing 5% BSA for 1 hour at room temperature. The bands were visualized by chemiluminescence analysis (GE Healthcare, Piscataway, NJ). Data processing was performed using a luminescent image analyzer LAS-3000 (FujiFilm, Tokyo, Japan) and IMAGE GAUGE software.

**LC-MS/MS analysis of SLs**

Ceramides were extracted using 50 µL of cell lysate (50 μg) or 10 mg tissue. 100 pM of internal standard C17-ceramide (d17:1/C18:0), and 2 mL of chloroform/methanol (2:1, v/v). Sphingosine, S1P or sphinganine were extracted with the mixture of 50 µL cell lysates (50 μg) or 10 mg tissue, 100 pM of internal standard C_17_-sphingosine and C_17_-S1P, and 1.2 mL of chloroform/methanol (2:1, v/v) solution and 0.3 mL of 0.1 N HCl. The lower phase was collected after repeated extraction and dried under a vacuum.

The residue was re-dissolved in 100 μL methanol, and then 10 μL was injected into the LC-ESI-MS/MS machine. For optimization, a mixture of ceramide standards or S1P and sphingosine was infused directly into the mass spectrometer and all source parameters and ionization conditions were adjusted to improve the sensitivity of the assay. Extracted samples (10 μL) were injected into a HPLC machine (Agilent 1200 series, Agilent, CA) and separated by reverse phase KINETEX C18 column chromatography (2.1 × 50 mm, ID: 2.6 μm) (Phenomenex, St. Louis, MO).

Ceramides were resolved using a linear gradient starting from 8% mobile phase A (water containing 0.2% formic acid) at a flow rate of 0.3 mL/min for 1 min, to 100% mobile phase B (methanol containing 0.2% formic acid) over 3 min, followed by 100% mobile phase B for 12 min. The column was then equilibrated for 8 min with 92% mobile phase B.

Sphingosine, S1P and sphinganine were resolved using a linear gradient from 50% mobile phase A (water containing 0.2% formic acid with 1 mM ammonium formate) at a flow rate of 0.3 mL/min for 1 min, to 100% mobile phase B (methanol containing 0.2% formic acid with 1 mM ammonium formate) over 3 min, followed by 100% mobile phase B for 8 min. The column was then equilibrated for 7 min with 50% mobile phase B.

The HPLC column effluent was introduced into an API 3200 Triple quadrupole mass spectrometer (ABCIEX, Toronto, Canada) and analyzed using electrospray ionization in positive mode. Analyses were performed using electrospray ionization in the positive-ion mode with multiple reactions monitoring to select both parent and characteristic daughter ions specific to each analyte simultaneously from a single injection. The MS/MS transitions (m/z) were 510→264 for C14, 538→264 for C16, 552→250 for C17, 566→264 for C18, 594→264 for C20, 648→264 for C24:1, 650→264 for C24, 366→250 for C_17_-S1P, 380→264 for C_18_-S1P, 286→238 for C_17_-sphingosine, 300→252 for C_18_-sphingosine, 302→60 for C_18_-sphinganine. Data were acquired using Analyst 1.4.2 software.

**Immunostaining**

For immunohistochemical examination, the stomach was removed and fixed by immersion in a periodate-lysine-2% paraformaldehyde solution overnight at 4°C. Tissues were cut transversely into 1 to 2 mm-thick slices, which were embedded in paraffin. Four micrometer sections were made, deparaffinized with xylene, and hydrated in a graded series of ethanol. After antigen retrieval, K_Ca_1.1 and phosphorylated myosin light chain (p-MLC) were identified in the tissues by using the respective primary and fluorescence-tagged secondary antibodies. Fluorescence images were acquired by confocal laser microscopy.

**References**

Nilius B, Schwartz G, Oike M, Droogmans G (1993). Histamine-activated, non-selective cation currents and Ca2+ transients in endothelial cells from human umbilical vein. *Pflugers Arch*. **424**, 285-293.

**Supplementary Figures**

**Fig. S1. Changes in levels of CerS and SLs in gastric SMCs by CerS2 ablation.**

**(A and B)** Levels of CerS1-CerS6 mRNA **(A)** and the levels of ceramides with various acyl chain lengths **(B)** were measured in gastric SMCs from 25-week-old CerS2-null and age-matched WT mice. Blots are representative of 3 experiments and results have been normalized to GAPDH levels. The data are quantified from a set of 3 experiments. **P* < 0.05, ***P* < 0.01 versus WT.


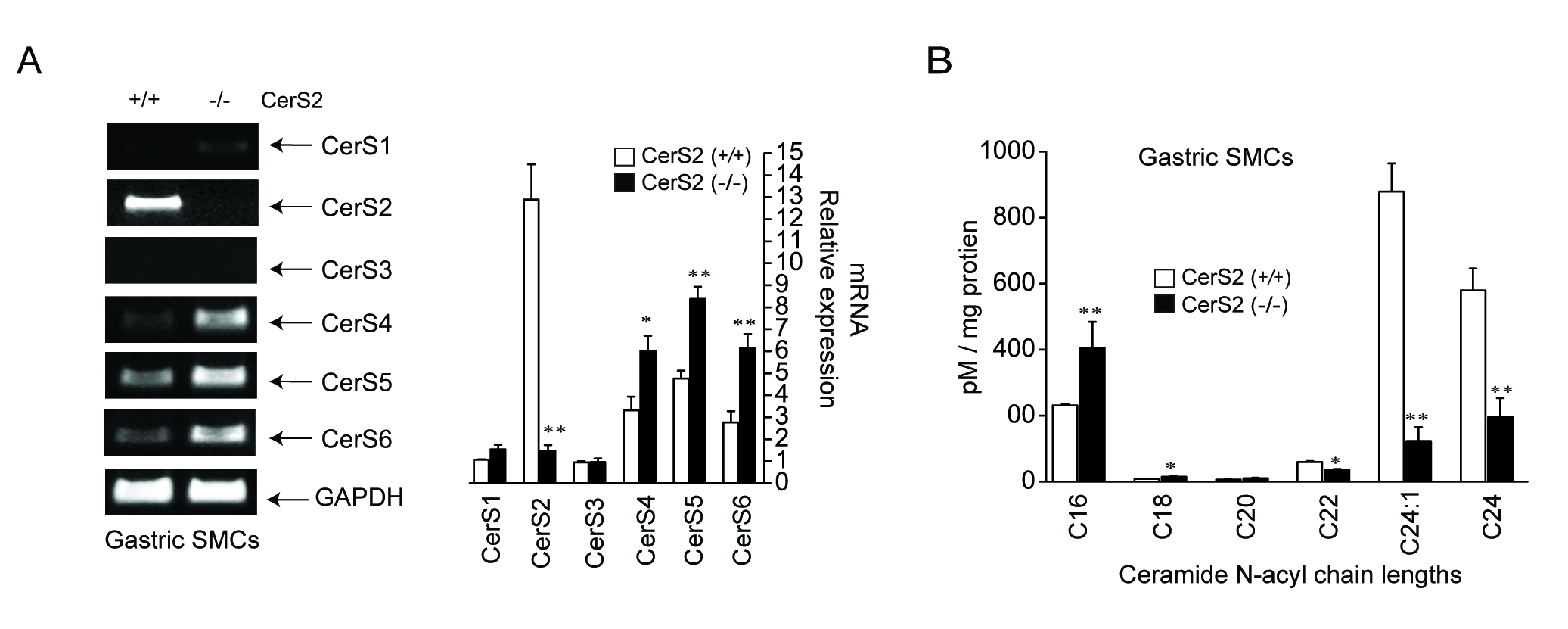


**Fig. S2. K_Ca_1.1 levels in primary cultured gastric SMCs from CerS2-null mice.**

mRNA **(A)** and protein **(B)** levels of K_Ca_1.1 were examined in primary cultured gastric SMCs from 25-week-old CerS2-null and age-matched WT mice. Blots are representatives of 4 experiments. Results were normalized to GAPDH or α-tubulin levels. **P* < 0.05, ***P* < 0.01 versus 25-week-old WT.


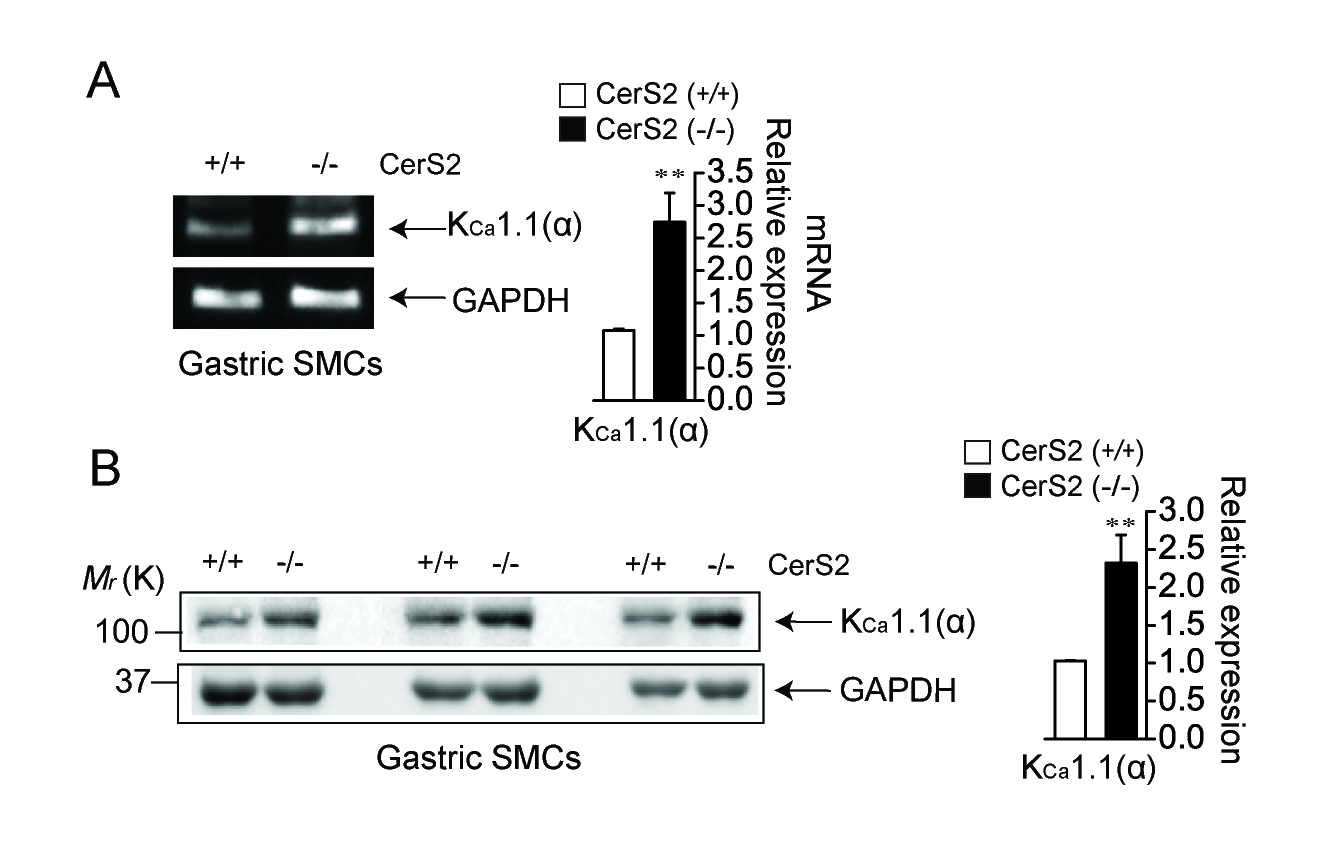


**Fig. S3. Changes in levels of ceramides with various acyl chain lengths by CerS5 transfection or CerS2 knock-down.**

**(A - D)** mRNA expressions of CerS1-CerS6 **(A and C)** and the levels of ceramides with various acyl chain lengths **(B and D)** were measured in WT primary gastric SMCs after transfection with CerS5 **(A and B)** or treatment with siRNA against CerS2 **(C and D)** for 24 hours. Blots are representative of 3 experiments and results have been normalized to GAPDH levels. The data are quantified from a set of 3 experiments. ***P* < 0.01 versus WT.


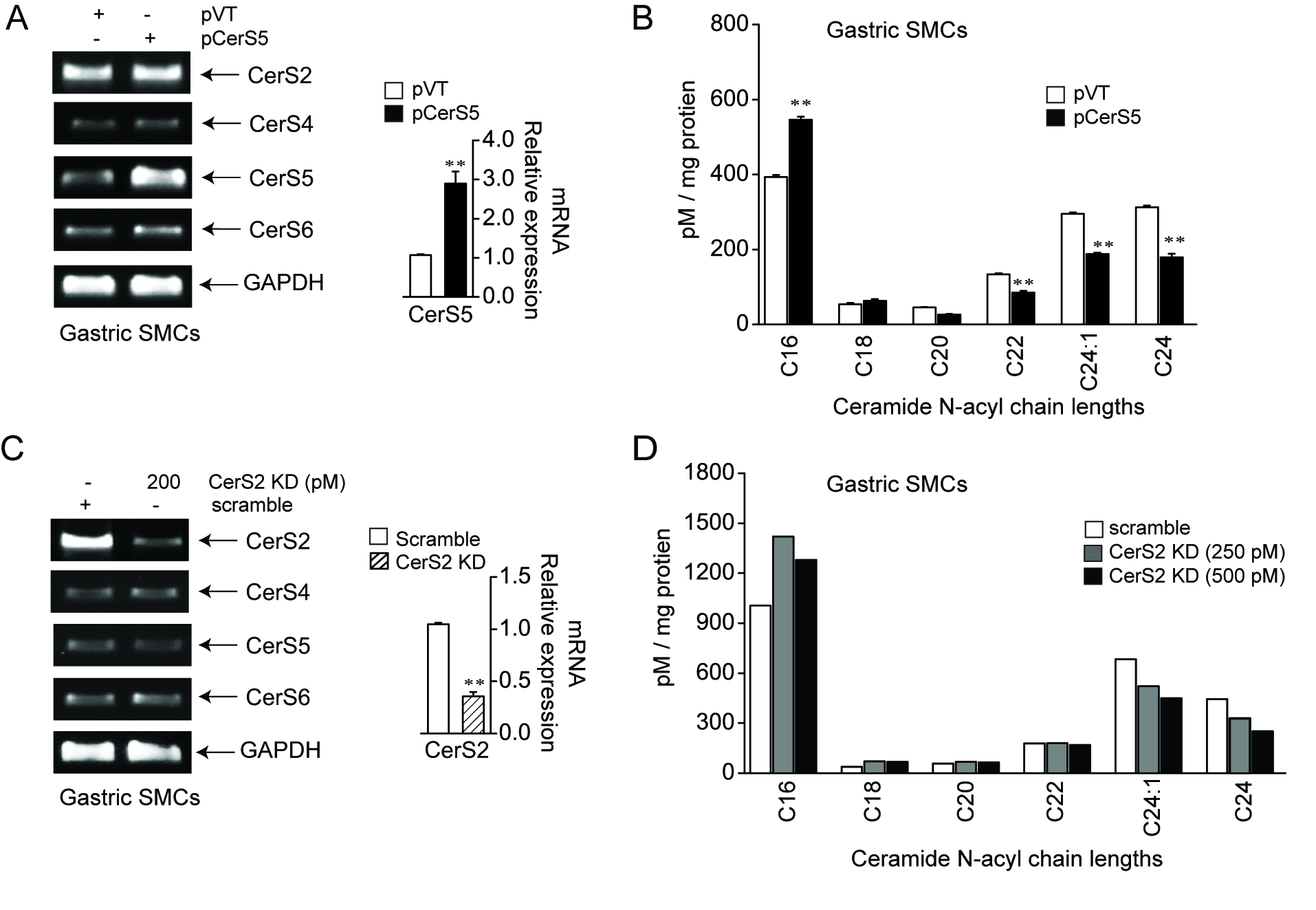


**Fig. S4. Inverse relationship between expression levels of K_Ca_1.1 and p-MLC.**

**(A)** Expression levels of K_Ca_1.1 and p-MLC in gastric smooth muscle tissues from 25-week-old CerS2-null (lower panel) and age-matched WT (upper panel) mice. The labeled K_Ca_1.1 and p-MLC were detected using an Alexa Fluor 488-conjugated secondary antibody (green), and Alexa Fluor 555-conjugated secondary antibody (red), respectively. **(B)** A higher magnification image of the boxed area is displayed. Nuclei were stained with DAPI (blue). Similar results were observed in each set of 3 experiments. Scale bar: 5 μm.


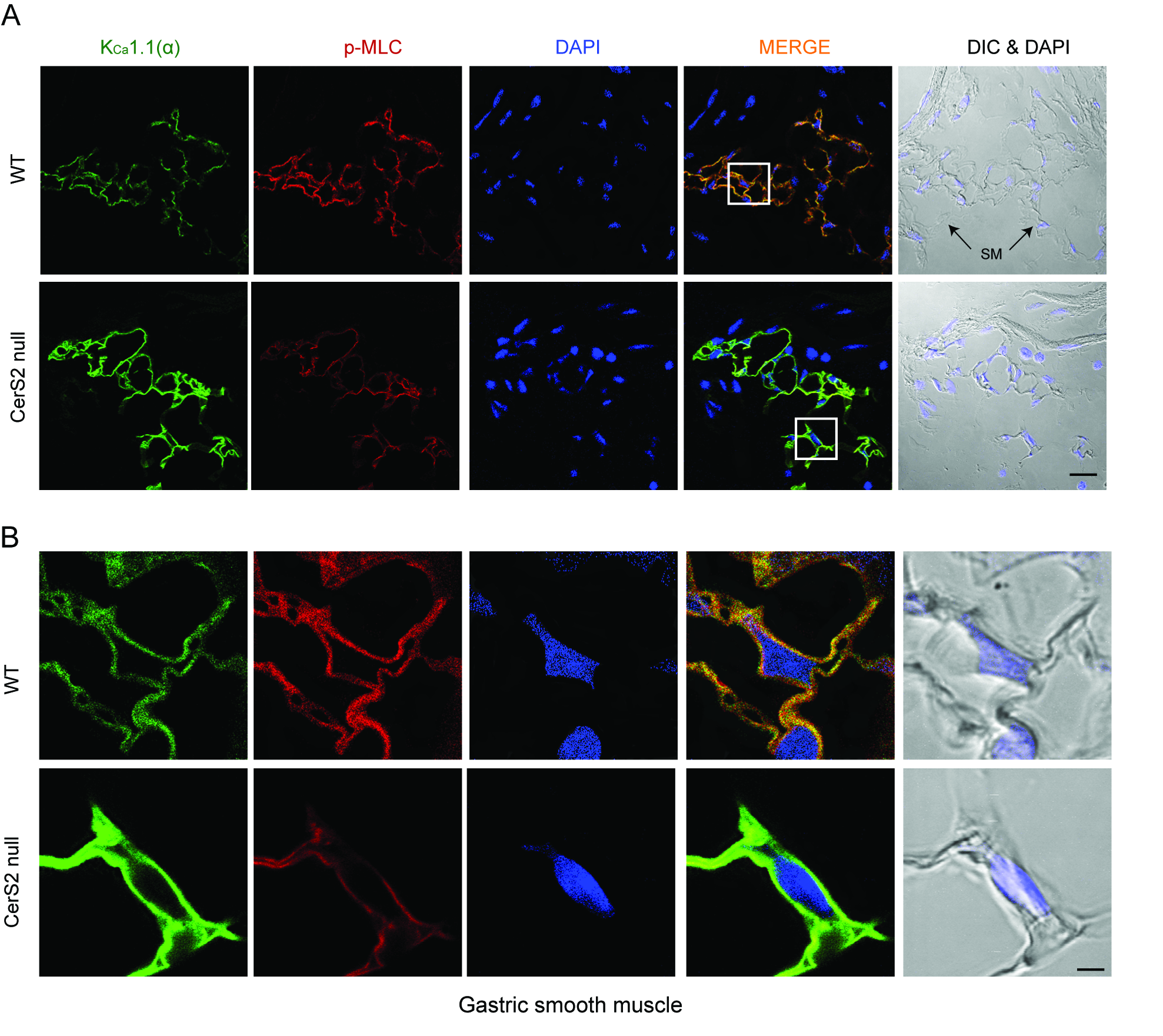


**Fig. S5. Tetrodotoxin did not prevent contractile dysfunction of aged WT or young CerS2-null gastric smooth muscle.**

**(A-C)** ACh-induced contraction of gastric smooth muscle from young WT **(A)**, young CerS2-null **(B)**, and aged WT **(C)** mice in the presence of tetrodotoxin (1 μM). The K_Ca_1.1 blocker, IBTx, recovered ACh-induced contractile dysfunction of gastric smooth muscle from young CerS2-null and aged WT mice.


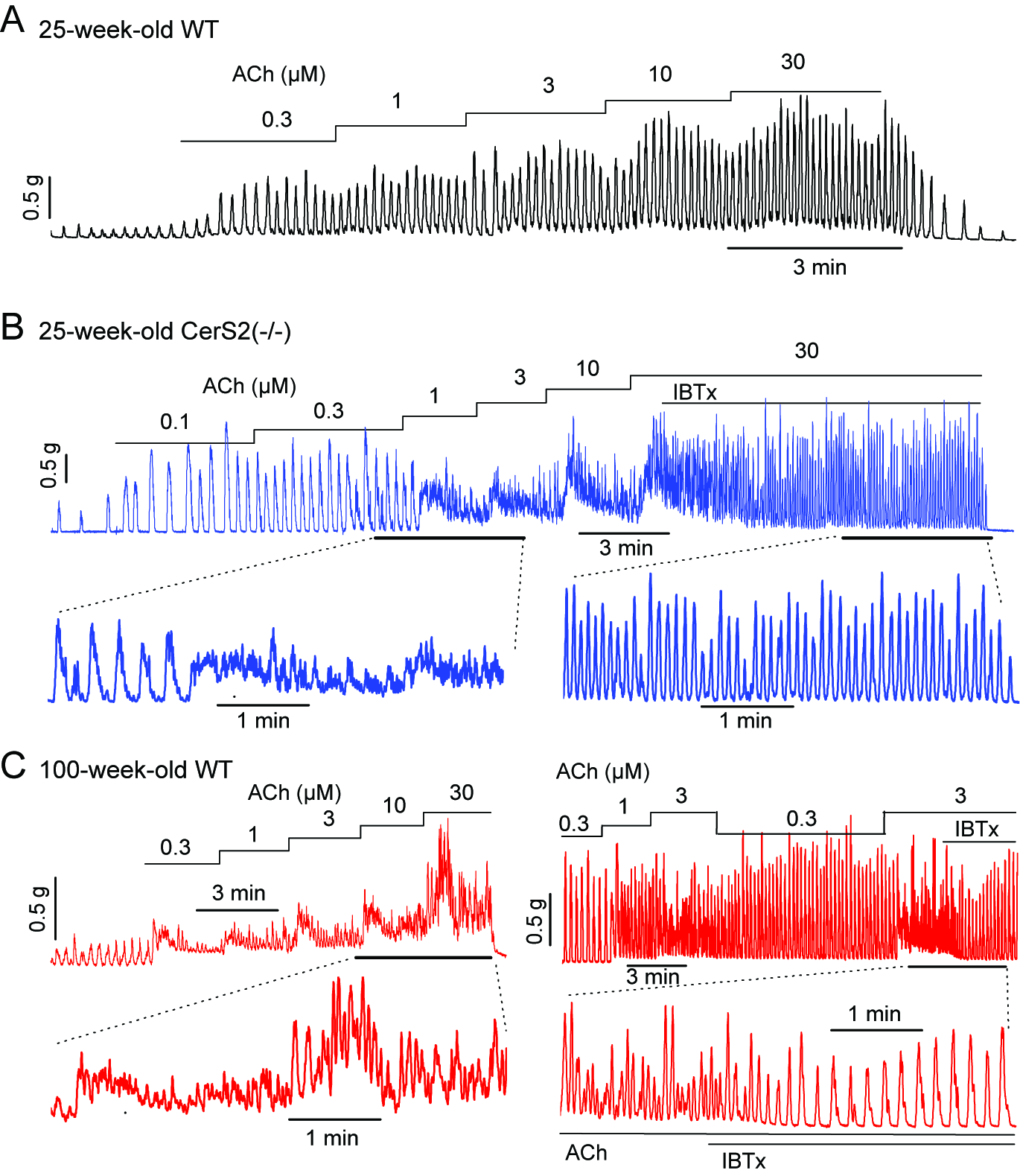


**Fig. S6. p21*^CIP1^* upregulation in gastric smooth muscle from aged WT and CerS2-null mice.**

**(A)** Protein levels of K_Ca_1.1 and p21*^CIP1^* were upregulated in an age-dependent manner in gastric smooth muscle from WT mice at different ages. mRNA **(B)** or protein **(C)** levels of p21*^CIP1^* in gastric smooth muscle or gastric SMCs from 25-week-old CerS2-null and age-matched WT mice. Blots are representative of 3-4 experiments. Results are normalized to GAPDH levels. **P* < 0.05, ***P* < 0.01 versus control (white column).


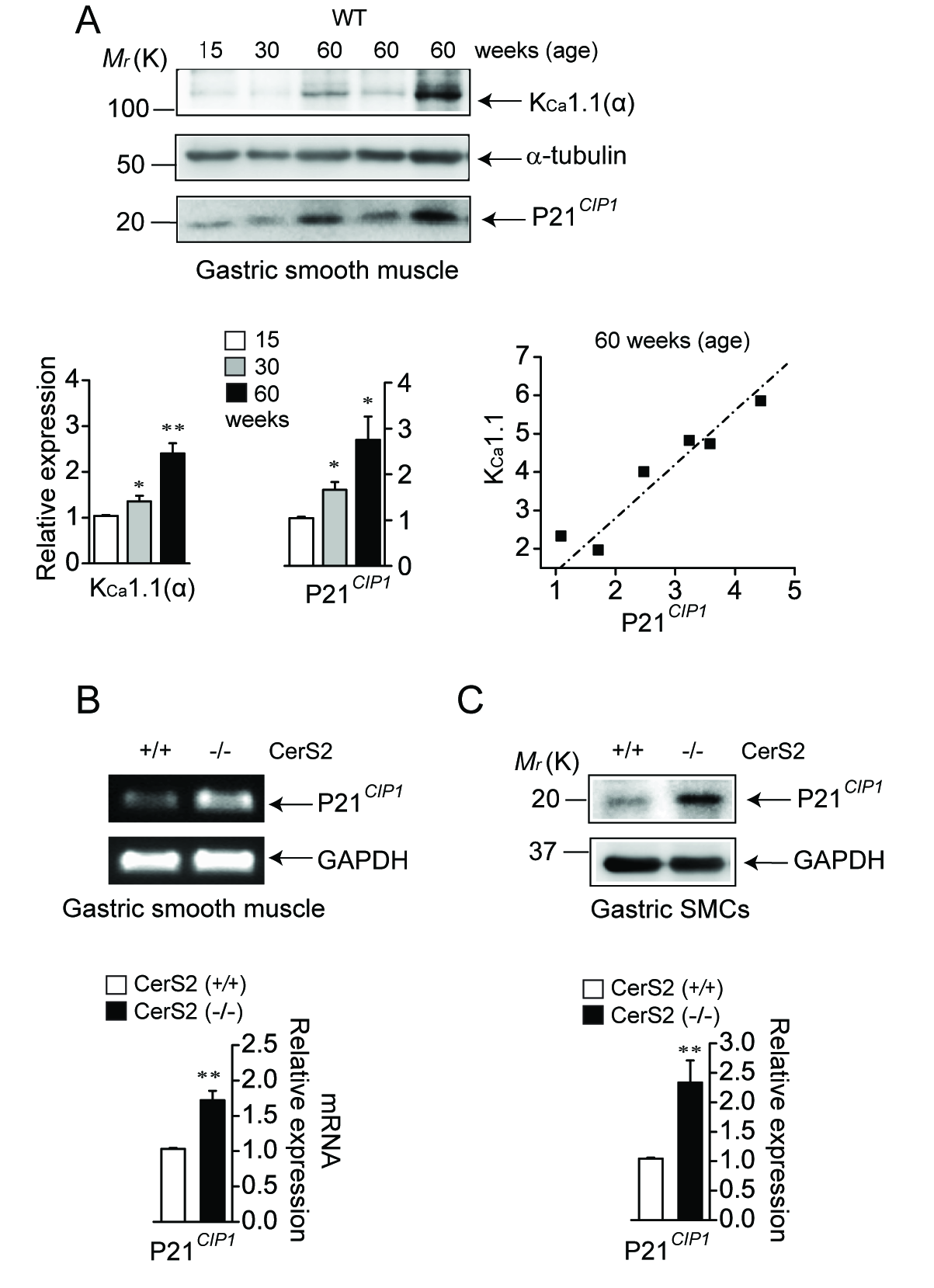

Supplement: Supplementary file 1 — Appendix S1. Supplementary Materials and methods. Fig. S1 Changes in levels of CerS and SLs in gastric SMCs by CerS2 ablation. Fig. S2 K Ca1.1 levels in primary cultured gastric SMCs from CerS2‐null mice. Fig. S3 Changes in levels of ceramides with various acyl chain lengths by CerS5 transfection or CerS2 knock‐down. Fig. S4 Inverse relationship between expression levels of K Ca1.1 and p‐MLC. Fig. S5 Tetrodotoxin did not prevent contractile dysfunction of aged WT or young CerS2‐null gastric smooth muscle. Fig. S6 p21CIP1 upregulation in gastric smooth muscle from aged WT and CerS2‐null mice. [file ACEL-14-0982-s001.docx]
